# Effects of a single tDCS with mirror therapy stimulation on hand function in healthy individuals

**DOI:** 10.3389/fnhum.2025.1607022

**Published:** 2025-06-18

**Authors:** Małgorzata Wójcik, Přemysl Vlček, Idzi Siatkowski, Marcela Grünerová-Lippertová

**Affiliations:** ^1^Department of Physiotherapy, Faculty of Sport Sciences in Gorzow Wielkopolski, Poznan University of Physical Education, Poznań, Poland; ^2^Third Faculty of Medicine, Charles University, Prague, Czechia; ^3^National Institute of Mental Health, Klecany, Czechia; ^4^Department of Mathematical and Statistical Methods, Poznan University of Life Science, Poznań, Poland; ^5^Clinic of Rehabilitation Medicine, University Hospital Královské Vinohrady, Prague, Czechia

**Keywords:** tDCS stimulation, mirror therapy, healthy people, hand, test

## Abstract

**Introduction:**

Transcranial direct current stimulation (tDCS) is a non-invasive neuromodulatory technique that has garnered significant interest for its ability to modulate cortical excitability and brain function. The technique involves the application of a weak electrical current through electrodes placed on the scalp, which influences neuronal membrane potential and alters synaptic plasticity.

**Methods:**

The following research hypotheses were defined: (1) a single tDCS stimulation of the motor cortex on the left side in combination with mirror therapy (MT) improves the function of the right hand which is dominant; and (2) a single stimulation in combination with MT for the dominant hand (right hand) also improves the function of the non-dominant hand (left hand). A total of 106 subjects aged 51.5 ± 12.02 participated in the study, including 63 women and 43 men. We used tests for assessment before and after tDCS stimulation: Tapping Tablet, Reaction Test on a tablet, Nine Hole Peg Test (NHPT), exercises for dominant hands, tDCS stimulation with MT.

**Results:**

The obtained results of this conducted study, although preliminary, seem to indicate that in each of the analyzed age groups in men and women, a single tDCS stimulation in combination with MT directly improved the function of the dominant hand and indirectly improved the function of the non-dominant hand.

**Discussion:**

The use of tDCS stimulation appears promising to improve hand function.

## 1 Introduction

Transcranial direct current stimulation (tDCS) is a non-invasive neuromodulatory technique that has garnered significant interest for its ability to modulate cortical excitability and brain function (van Velsen et al., 2013). The technique involves the application of a weak electrical current through electrodes placed on the scalp, which influences neuronal membrane potential and alters synaptic plasticity (Nitsche and Paulus, 2000). The effects of tDCS are polarity dependent: anodal stimulation, which delivers positive current, generally increases neuronal excitability by facilitating depolarization of the resting membrane potential, thus enhancing synaptic transmission and cortical excitability (Stagg et al., 2018; Walhovd et al., 2016). On the contrary, cathodal tDCS, which applies negative stimulation, is associated with hyperpolarization of the resting membrane potential, leading to suppression of neuronal excitability and cortical activity (Jacobson et al., 2021; Barbieri et al., 2016).

The effectiveness of tDCS-induced excitability changes is influenced by several factors, including the duration of stimulation, the intensity of the current, and individual neurophysiological characteristics (Paulus et al., 2016; Woods et al., 2016). Research suggests that prolonged stimulation may enhance long-term potentiation (LTP)-like effects, while shorter durations may only induce transient excitability changes (Agboada et al., 2020). Given its ability to modulate cortical activity, tDCS has emerged as a promising intervention in neuropsychiatric and neurological disorders. It has been increasingly used for the treatment of major depressive disorder, anxiety disorders, and drug-resistant schizophrenia, particularly to alleviate symptoms such as auditory hallucinations and cognitive deficits (Rodrigues et al., 2023; Lefaucheur et al., 2017; Fröhlich et al., 2016; Brunoni et al., 2016). Furthermore, ongoing research continues to explore its potential applications in neurorehabilitation, pain management, and cognitive enhancement in both clinical and healthy populations (Ciechanski et al., 2019; Bikson et al., 2018).

Given its ability to modulate neuroplasticity and cortical excitability, tDCS has been extensively investigated in motor rehabilitation and performance enhancement, particularly for its role in improving upper limb function (Claudia et al., 1981). Research in both clinical and healthy populations has explored how tDCS can enhance motor skill learning, promote recovery following neurological damage, and optimize dexterity and coordination (Siew-Pin Leuk et al., 2022; Pixa and Pollok, 2018; Buch et al., 2017; Kang et al., 2016; Goodwill et al., 2013). In neurorehabilitation, anodal tDCS over the primary motor cortex (M1) has shown promising results in facilitating motor recovery, particularly in stroke patients and individuals with motor impairment (Bornheim et al., 2022). Additionally, studies employing bihemispheric tDCS paradigms suggest that balancing interhemispheric interactions between motor cortices can further optimize upper limb function, supporting applications in both rehabilitation and performance improvement (Tazoe et al., 2014; Lefebvre et al., 2014).

One of the key mechanisms through which tDCS influences motor function is by modulating interhemispheric dynamics (Waters et al., 2017). The motor cortices in both hemispheres maintain reciprocal interactions, where excitatory and inhibitory processes contribute to coordinated movement control (MacDonald et al., 2021). tDCS can enhance cortical excitability in the stimulated hemisphere, while suppressing excessive interhemispheric inhibition, thus promoting neuroplastic changes that refine motor output and skill acquisition (Tazoe et al., 2014; Sehm et al., 2013; Vines et al., 2008).

In clinical populations, particularly stroke survivors and individuals with neurological impairments, combining tDCS with mirror therapy (MT) has shown synergistic benefits for motor recovery (Zhao et al., 2022). MT, which uses visual feedback to activate bilateral motor networks, is believed to enhance sensorimotor integration and facilitate neuroplasticity (Madhoun et al., 2020; Grunt et al., 2017). When paired with anodal tDCS over the motor cortex, MT has been found to improve hand function, increase movement precision, and accelerate rehabilitation outcomes (Liao et al., 2020).

Numerous studies have demonstrated that tDCS can enhance motor performance in healthy individuals by modulating cortical excitability and interhemispheric interactions. When anodal tDCS is applied over the primary motor cortex (M1), improvements in motor skill acquisition, reaction times, and upper limb strength have been observed in non-clinical populations (Hazime et al., 2017; Vargas et al., 2018). A systematic review and meta-analysis confirmed that tDCS significantly enhances upper limb coordination and dexterity, particularly when paired with motor training tasks (Patel et al., 2019). Furthermore, research examining fine motor control found that stimulation increased tapping speed and precision, reinforcing its role in improving motor execution (Edwards et al., 2017; Banissy and Muggleton, 2013). More recent studies suggest that tDCS-induced plasticity contributes to improved neuromuscular efficiency, leading to greater upper limb function (Park et al., 2023). These findings align with earlier work on interhemispheric asymmetry in motor control, which demonstrated that modulating excitability in the dominant motor cortex produces differential effects on ipsilateral and contralateral limb function (Vines et al., 2008).

Additional support for these effects comes from research on highly trained individuals, further reinforcing the role of tDCS in optimizing motor function. For example, the application of anodal tDCS to the primary motor cortex has been shown to enhance isometric strength in the shoulder rotator muscles of healthy subjects engaged in repetitive upper-limb tasks (Hazime et al., 2017). Similarly, stimulation has been associated with improvements in complex movement execution, with evidence showing enhanced neuromuscular coordination and efficiency (Park et al., 2023). Since athletes possess refined motor skills due to extensive practice, the observed enhancements suggest that the effects of tDCS extend beyond initial skill acquisition to optimizing motor networks that are already well trained. These findings collectively support the role of tDCS in motor enhancement, strengthening its potential applications in cognitive-motor training and neurorehabilitation.

Although accumulating evidence supports the beneficial effects of combining tDCS with motor tasks, research investigating the precise impact of anodal tDCS combined with MT on upper limb motor performance in healthy populations remains relatively limited. In the present study, we sought to examine whether the application of anodal tDCS over the primary motor cortex representing the dominant upper extremity, when integrated with MT, enhances motor performance of the dominant limb. Furthermore, we aimed to determine whether any observed motor improvements would also transfer to the non-dominant limb via interhemispheric mechanisms.

Before proceeding, the following research hypotheses were defined:

1. a single tDCS stimulation of the motor cortex on the left side in combination with MT improves the function of the right hand that is dominant;
2. a single stimulation in combination with MT for the dominant hand (right hand) also improves the function of the non-dominant hand (left hand).

## 2 Materials and methods

### 2.1 Type of study and participants

This investigation was carried out as a single group repeated measures experimental study aimed at examining the effects of a single session anodal tDCS in healthy adults. A total of 106 right-handed volunteers (63 women and 43 men) participated, with an overall mean age of 51.5 ± 12.02 years. Participants were recruited from the local community through informational flyers and word of mouth. All individuals gave their informed consent in accordance with the Declaration of Helsinki before participating in any study procedures.

The eligibility criteria specified that participants must be right-handed, between 40 and 70 years of age, without a history of neurological or rheumatic disorders, upper limb fractures, or recent soft-tissue injuries. Additional exclusion criteria included active alcohol consumption, tobacco use, or any contraindications to tDCS. Individuals were informed of their right to withdraw at any point without penalty. The study protocol was approved by the Institutional Ethics Committee (permit No. 20/25, 9 January 2025), and the trial was registered under NCT06891690.

### 2.2 Test battery and protocol

To evaluate the effects of tDCS stimulation combined with MT on hand function, we employed a structured battery of motor function tests. These included the Nine Hole Peg Test (NHPT), the Click Speed Test, and the Flipflop Reflex Test. The NHPT is a validated and widely used measure of fine motor coordination and manual dexterity in both clinical and healthy populations (Kellor et al., 1971; Mathiowetz et al., 1985; Vanbellingen et al., 2017; Allgöwer and Hermsdörfer, 2017; Jobbágy et al., 2018). The Click Speed and Flipflop Reflex tests, though less commonly cited in the literature, are freely available and offer accurate, time-based assessments of finger tapping speed and reaction time, respectively (Click Speed Test, 2025; Flipflop Reflex Test, 2025). The primary advantage of these tools lies in their high measurability, enabling objective comparison of motor performance before and after intervention.

The study took place between 10:00 am and 2:00 pm, in a quiet and warm room, in a sitting position. The same protocol was used for all subjects considering:

Tests to assess dominant and non-dominant hand function before tDCS stimulation:

1. Tapping tablet test, medium level (Click Speed Test, 2025), the duration of the test was 30 s, the time was measured by the device, the middle finger of the dominant hand was tested, the subject sat at a table, the forearms were rested on the table top, the tablet was in front of the test hand, the subject’s task was to perform tapping with the middle finger in 30 s trying to touch the tablet monitor with the finger as many times as possible,
2. Tapping test on a tablet, medium level (Click Speed Test, 2025), performance as above, the test involved the middle finger of the non-dominant hand,
3. Reaction test on a tablet, medium level (Flipflop Reflex Test, 2025), the index fingers of the dominant hand were tested, the test subject sat at a table, the forearms were rested on the table top, the tablet was in front of the test hand, the test subject’s task was to touch the shining point as quickly as possible (the test subject had a total of nine points to touch which were arranged in the form of a quadrilateral), after the test was completed, the device showed the time to complete the test,
4. Reaction tablet test, medium level (Flipflop Reflex Test, 2025), performance as above, the test involved the middle finger of the non-dominant hand,
5. Nine Hole Peg Test (Temporiti et al., 2023), the dominant hand’s grasping function was tested, the subject sat at a table, his forearms were rested on the table top, the subject’s task was to place a peg in a hole in the shortest possible time (there were nine pegs and holes), then the subject had to remove each peg and put it aside, the time was measured with a stopwatch, when testing the dominant hand, the holes for placing the pegs were in front of the subject and the pegs were on his left side,
6. Nine Hole Peg Test dominant hand (Temporiti et al., 2023), the non-dominant hand was tested as above, when testing the non-dominant hand, the holes for placing the pegs were in front of the subject and the pegs were on his right side. Mathiowetz et al. (1985) reported that healthy adult men completed the NHPT in an average of 19.0 s with the right hand and 20.6 s with the left hand.

Then, after hand testing, tDCS stimulation was proceeded, the area of the electrodes was 25 cm^2^, the anode was placed on C3 and the cathode was placed on Fp2, the maximum intensity value was 2 mA and the duration of stimulation was 20 min, the stimulation duration of stimulation was measured by a timer built into the device. After 10 min of stimulation, the subject performed MT for the dominant hand. During MT, the right (dominant) limb was rested on the table top, the subject’s task was to make movements with the hand by turning on the gaze (the subject made movements with the hand by seeing them in the mirror); when performing MT, care was taken that the subject only saw the reflection of his hand in the mirror. MT consisted of the following hand movements at a specific time, which was measured by a stopwatch:

1. wrist extension for 1 min (forearm was rested on the table top in an intermediate position),
2. 30 s rest,
3. wrist flexion for 1 min (the forearm was rested on the table top in an intermediate position),
4. 30 s rest,
5. flexion and extension of the fingers for 1 min (the forearm was rested on the table top in a pronated position),
6. 30 s rest,
7. clenching and opening fists for 1 min (the forearm was rested on the table top in an intermediate position),
8. 30 s rest,
9. inversion and adduction of the fingers for 1 min (the forearm was rested on the table top in a pronated position),
10. 30 s rest,
11. tapping with the middle finger for 1 min (the forearm was rested on the table top in a pronated position),
12. 30 s rest,
13. tapping with all fingers for 1 min (the forearm was rested on the table top in a pronated position),
14. 30 s rest,

After completion of MT, tests were repeated to assess dominant and non-dominant hand function. The time values were recorded for each participant before and after tDCS stimulation. During and after tDCS stimulation, participants reported no side effects.

### 2.3 Statistical analyses

Before proceeding to static analysis, the Shapiro–Wilk test was applied to check the data distributions. It was observed that the condition of normal distribution was not met. Therefore, the Wilcoxon signed-rank test with continuity correction was used to analyze two sets of paired data (sample before vs. sample after) and unpaired data (sample before vs. before or sample after vs. after), while the Kruskal–Wallis test and Dunn test with Bonferroni correction were used to analyze four sets of data. The results are presented in tables and figures. The R statistical package (R Core Team, 2024) was used for statistical analysis. Statistical analysis was performed taking into account the division into groups: all subjects, women and men, and the division by age. Since the Kruskal–Wallis test and Dunn’s test are applied to independent groups (not paired), only statistically significant differences of independent groups are indicated in the figures.

## 3 Results

**FIGURE 1:**
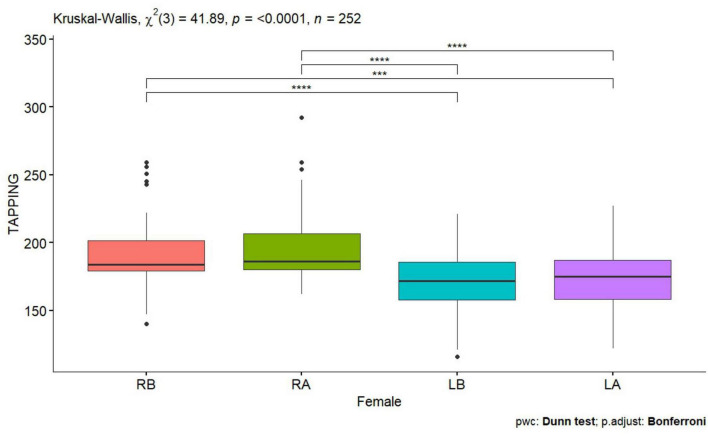
Tapping test of a woman before and after performing tDCS stimulation with mirror therapy for the right and left hand [RB, Right Before stimulation tDCS (right hand); RA, Right After stimulation tDCS (left hand); LB, Left Before stimulation tDCS (left hand); LA, Left After stimulation (left hand)]. Only statistically significant differences for independent groups are indicated, where ***p-value < 0.001; ****p-value < 0.0001.

**FIGURE 2:**
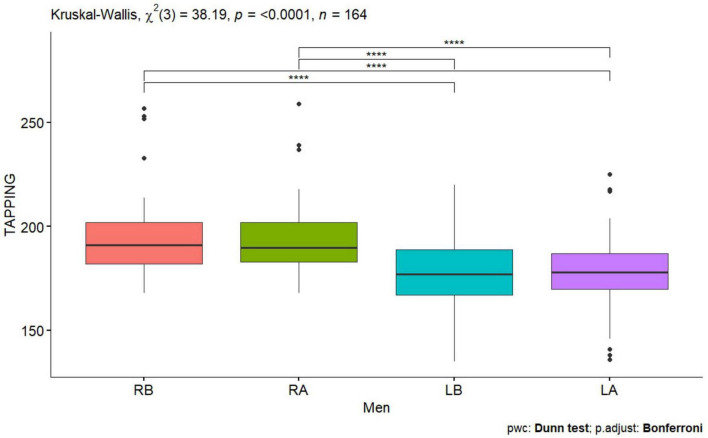
Tapping test men before and after performing tDCS stimulation with mirror therapy for the right and left hand [RB, Right Before stimulation tDCS (right hand); RA, Right After stimulation tDCS (left hand); LB, Left Before stimulation tDCS (left hand); LA, Left After stimulation (left hand)]. Only statistically significant differences for independent groups are indicated, where ****p-value < 0.0001.

**FIGURE 3:**
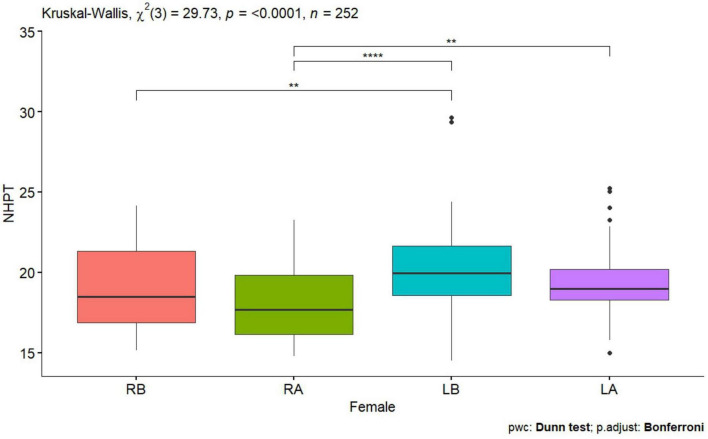
Nine Hole Peg Test of a woman before and after tDCS stimulation with mirror therapy for the right and left hand [RB, Right Before stimulation tDCS (right hand); RA, Right After stimulation tDCS (left hand); LB, Left Before stimulation tDCS (left hand); LA, Left After stimulation (left hand)]. Only statistically significant differences for independent groups are indicated, where **p-value < 0.01; ****p-value < 0.0001.

**FIGURE 4:**
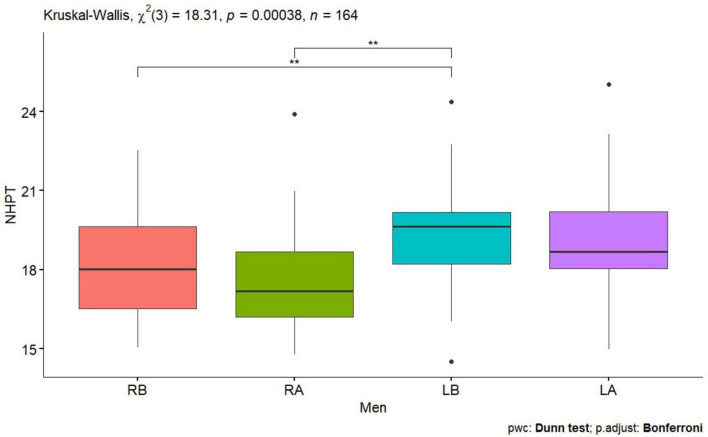
Nine Hole Peg Test men before and after tDCS stimulation with mirror therapy for the right and left hands [RB, Right Before stimulation tDCS (right hand); RA, Right After stimulation tDCS (left hand); LB, Left Before stimulation tDCS (left hand); LA, Left after stimulation (left hand)]. Only statistically significant differences for independent groups are indicated, where **p-value < 0.01.

**FIGURE 5:**
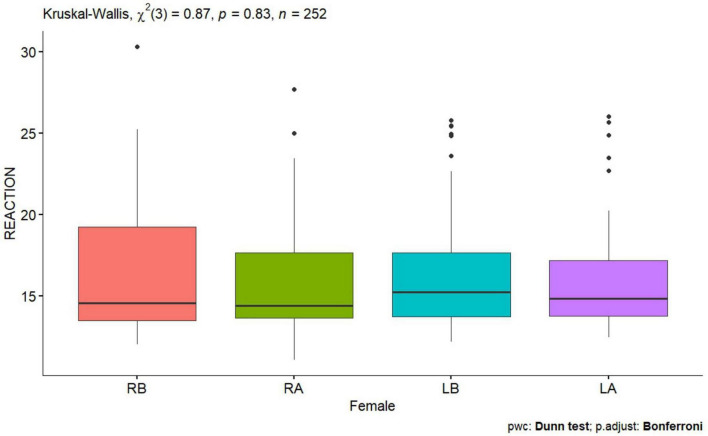
Reaction test of a woman before and after tDCS stimulation with mirror therapy for the right and left hand [RB, Right Before stimulation tDCS (right hand); RA, Right After stimulation tDCS (left hand); LB, Left Before stimulation tDCS (left hand); LA, Left After stimulation (left hand)].

**FIGURE 6:**
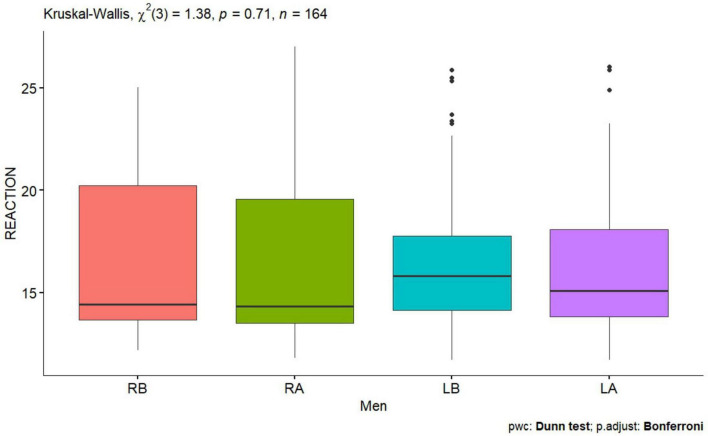
Reaction test men before and after performing tDCS stimulation with mirror therapy for the right and left hand [RB, Right Before stimulation tDCS (right hand); RA, Right After stimulation tDCS (left hand); LB, Left Before stimulation tDCS (left hand); LA, Left After stimulation (left hand)].

First, statistical analysis was performed for the data obtained from the Tapping, Reaction and Nine Hole Peg Test (NHPT) before and after tDCS stimulation in all subjects and taking into account the division into women and men separately (Table 1 and Figures 1–6).

**TABLE 1:** Wilcoxon test: analysis of the results of the Tapping, Reaction, and Nine Hole Peg Test (NHPT) before and after tDCS stimulation for the dominant hand, i.e., right hand and non-dominant left hand.

		p-Values		
**Comparison**	**Wilcoxon test**	**All**	**Women**	**Men**
TAPPING_RB vs. TAPPING_RA	Paired	0.0003	0.0001	0.3405
REACTION_RB vs. REACTION_RA	Paired	<0.0001	<0.0001	0.0009
NHPT_RB vs. NHPT_RA	Paired	<0.0001	0.0002	0.0002
TAPPING_LB vs. TAPPING_LA	Paired	0.0637	0.0747	0.3789
REACTION_LB vs. REACTION_LA	Paired	0.0539	0.2902	0.0256
NHPT_LB vs. NHPT_LA	Paired	0.0277	0.0090	0.6442
TAPPING_RB vs. TAPPING_LB	Unpaired	<0.0001	< 0.0001	<0.0001
REACTION_RB vs. REACTION_LB	Unpaired	0.5709	0.7809	0.5486
NHPT_RB vs. NHPT_LB	Unpaired	<0.0001	0.0008	0.0086
TAPPING_RA vs. TAPPING_LA	Unpaired	<0.0001	< 0.0001	<0.0001
REACTION_RA vs. REACTION_LA	Unpaired	0.3733	0.6221	0.4360
NHPT_RA vs. NHPT_LA	Unpaired	<0.0001	0.0008	0.0011

RB, Right Before stimulation tDCS (right hand); RA, Right After stimulation tDCS (left hand); LB, Left Before stimulation tDCS (left hand); LA, Left After stimulation (left hand).

For the right hand for the Tapping test, statistical significance was observed before and after tDCS stimulation in all subjects *p* = 0.0003 (Table 1) and in women *p* = 0.0001 (Table 1). For the Reaction test, statistical significance was observed in all subjects *p* < 0.0001 (Table 1), in women *p* < 0.0001 (Table 1) and in men *p* = 0.0009 (Table 1).

Also, for the NHPT, statistically significant values were obtained in all subjects *p* < 0.0001 (Table 1), in men and women *p* = 0.0002 (Table 1). The results obtained indicate that single tDCS stimulation combined with mirror therapy improved the results of tests assessing hand function.

For the left hand for the Reaction test, statistical significance was observed in men *p* = 0.0256 (Table 1), for the NHPT statistical significance was recorded for all *p* = 0.0277 (Table 1) and for women *p* = 0.0090 (Table 1) and for the Tapping test statistical significance was observed *p* < 0.0001 in all subjects, women and men (Table 1). The extracted values indicate that tDCS stimulation combined with mirror therapy indirectly influenced the values of hand function tests.

Statistically significant values were also observed for the right and left hand for the Tapping test before performing tDCS stimulation in all subjects, in women and men *p* < 0.0001 (Table 1 and Figures 1, 2), also for the NHPT significant statistical values were recorded in all subjects *p* < 0.0001 (Table 1), in women *p* = 0.0008 (Table 1 and Figure 3) and in men *p* = 0.0086 (Table 1 and Figure 4). Significant statistical values indicate that subjects had similar values for the right and left hands for tests to assess hand function before tDCS stimulation and mirror training.

After tDCS stimulation combined with mirror training, there was a statistical significant of *p* < 0.0001 (Table 1) in all subjects, in women and men for the Tapping test and for the NHPT in all subjects *p* < 0.0001 (Table 1), in women *p* = 0.0008 (Table 1 and Figures 1, 3) and in men (Table 1 and Figures 2, 4). These values indicate that similar values for tests assessing hand function were obtained for the right and left hands, indicating a direct effect of tDCs stimulation combined with mirror training on the functions of the dominant hand and indirect functions of the non-dominant hand.

No statistically significant difference was observed for the right and left hand before and after tDCS stimulation with mirror therapy in women group and man group (Figures 5, 6).

The next step was a statistical analysis for subjects aged 40–49, taking into account the analysis for the whole group and with a division into a group of men and women (Table 2).

**TABLE 2:** Wilcoxon test: analysis for the subjects’ age range of 40–49 years for the results of the Tapping, Reaction, Nine Hole Peg Test (NHPT) before and after tDCS stimulation for the dominant hand, i.e., right hand and non-dominant left hand.

		p-Values		
**Comparison**	**Wilcoxon test**	**All**	**Women**	**Men**
TAPPING_RB vs. TAPPING_RA	Paired	0.0019	0.0032	0.1970
REACTION_RB vs. REACTION_RA	Paired	<0.0001	0.0027	0.0004
NHPT_RB vs. NHPT_RA	Paired	<0.0001	0.0013	0.0212
TAPPING_LB vs. TAPPING_LA	Paired	0.0885	0.2040	0.2776
REACTION_LB vs. REACTION_LA	Paired	0.2616	0.7607	0.1289
NHPT_LB vs. NHPT_LA	Paired	0.2451	0.1257	0.8987
TAPPING_RB vs. TAPPING_LB	Unpaired	0.0001	0.0045	0.0048
REACTION_RB vs. REACTION_LB	Unpaired	0.5444	0.5050	0.8253
NHPT_RB vs. NHPT_LB	Unpaired	0.0004	0.0038	0.0346
TAPPING_RA vs. TAPPING_LA	Unpaired	<0.0001	0.0012	0.0048
REACTION_RA vs. REACTION_LA	Unpaired	0.2243	0.3013	0.5531
NHPT_RA vs. NHPT_LA	Unpaired	<0.0001	0.0004	0.0089

RB, Right Before stimulation tDCS (right hand); RA, Right After stimulation tDCS (left hand); LB, Left Before stimulation tDCS (left hand); LA, Left After stimulation (left hand).

For the right (dominant) hand after the application of tDCS stimulation combined with mirror therapy, statistically significant values were recorded for the following tests: Tapping in all subjects *p* = 0.0019 and women *p* = 0.0032 (Table 2); reaction in all subjects *p* < 0.0001, in women *p* = 0. 0027 and men *p* = 0.0004 (Table 2); NHPT also recorded statistical significance in all subjects, that is, all *p* < 0.0001, women *p* = 0.0013, and men *p* = 0.0212. These values indicate that tDCS stimulation combined with mirror therapy improved hand function (Table 2).

Before tDCS stimulation with mirror therapy, statistically significant values were recorded for the Tapping test for the right and left hands: all *p* = 0.0001, women *p* = 0.0045, and men *p* = 0.0048 (Table 2), indicating that the test values for both hands were similar.

After performing tDCS stimulation with mirror therapy, statistical significance was observed for the dominant and non-dominant hand for the Tapping test in subjects: all *p* < 0.0001, women *p* = 0.0012, and men *p* = 0.0048 (Table 2) and for the NHPT in subjects: all *p* < 0.0001, women *p* = 0.0004, and men *p* = 0.0089 (Table 2). These values say that after performing motor cortex stimulation for the dominant hand, both the dominant and non-dominant hand had similar values for tests assessing hand function.

The next step in the statistical analysis was to analyze the data of people aged 50–59, also taking into account the breakdown: the whole group, women and men (Table 3).

**TABLE 3:** Wilcoxon test: analysis for the age range of subjects 50–59 years for the results of the Tapping, Reaction, Nine Hole Peg Test (NHPT) before and after tDCS stimulation for the dominant hand, i.e., right hand and non-dominant left hand.

		p-Values		
**Comparison**	**Wilcoxon test**	**All**	**Women**	**Men**
TAPPING_RB vs. TAPPING_RA	Paired	0.0996	0.0965	0.6224
REACTION_RB vs. REACTION_RA	Paired	0.0070	0.0181	0.1591
NHPT_RB vs. NHPT_RA	Paired	0.0045	0.1819	0.0063
TAPPING_LB vs. TAPPING_LA	Paired	0.0955	0.0669	0.7971
REACTION_LB vs. REACTION_LA	Paired	0.1073	0.5412	0.0335
NHPT_LB vs. NHPT_LA	Paired	0.0043	0.0071	0.1928
TAPPING_RB vs. TAPPING_LB	Unpaired	<0.0001	0.0004	0.0007
REACTION_RB vs. REACTION_LB	Unpaired	0.9163	0.9081	0.7240
NHPT_RB vs. NHPT_LB	Unpaired	0.0064	0.0166	0.2204
TAPPING_RA vs. TAPPING_LA	Unpaired	<0.0001	0.0023	0.0014
REACTION_RA vs. REACTION_LA	Unpaired	0.9065	0.9070	0.6420
NHPT_RA vs. NHPT_LA	Unpaired	0.0660	0.2799	0.1713

RB, Right Before stimulation tDCS (right hand); RA, Right After stimulation tDCS (left hand); LB, Left Before stimulation tDCS (left hand); LA, Left After stimulation (left hand).

For the right hand (dominant), statistically significant values were observed for the Tapping test in all subjects *p* = 0.0070 (Table 3) and in women *p* = 0.0181 (Table 3). And for the NHPT in all *p* = 0.0045 and in men *p* = 0.0063 (Table 3). The results obtained indicate that the applied tDCS stimulation in combination with mirror therapy positively improved the function of the dominant hand. Also, for the non-dominant (left) hand, a statistically significant result was observed for the NHPT in all *p* = 0.0043 (Table 3) and women *p* = 0.0071 (Table 3). This result indicates that stimulation indirectly affected the function of the left hand.

Before stimulation of tDCS and mirror therapy, statistically significant values were observed for the dominant and non-dominant hand for the Tapping test for all *p* < 0.0001 (Table 3), in women *p* = 0.0004 (Table 3) and men *p* = 0.0007 (Table 3), and for the NHPT for all *p* = 0.0064 (Table 3) and women *p* = 0.0166 (Table 3), meaning that the values of the tests assessing hand function were similar.

Transcranial direct current stimulation combined with mirror therapy, statistically significant values were recorded for the right and left hands in the Tapping test *p* < 0.0001 (Table 3), in women *p* = 0.0023 (Table 3) and in men *p* = 0.0014 (Table 3), indicating that tDCS stimulation and mirror therapy directly affected the function of the right hand and indirectly of the left hand.

The final stage of the statistical study was the analysis for the 60–70 age range, considering the analysis for all subjects, separately for women and for men only (Table 4).

**TABLE 4:** Wilcoxon test: analysis for the subjects’ age range of 60–70 years for the results of the Tapping, Reaction, Nine Hole Peg Test (NHPT) before and after tDCS stimulation for the dominant hand, i.e., right hand and non-dominant left hand.

		*p*-Values		
**Comparison**	**Wilcoxon test**	**All**	**Women**	**Men**
TAPPING_RB vs. TAPPING_RA	Paired	0.3722	0.1056	0.5862
REACTION_RB vs. REACTION_RA	Paired	0.0371	0.0313	1
NHPT_RB vs. NHPT_RA	Paired	0.0273	0.1563	0.2500
TAPPING_LB vs. TAPPING_LA	Paired	0.5406	0.8438	0.7500
REACTION_LB vs. REACTION_LA	Paired	0.3223	0.1563	1
NHPT_LB vs. NHPT_LA	Paired	0.4752	1	0.5000
TAPPING_RB vs. TAPPING_LB	Unpaired	0.2408	0.3358	0.4000
REACTION_RB vs. REACTION_LB	Unpaired	0.7394	0.6991	0.2000
NHPT_RB vs. NHPT_LB	Unpaired	0.5196	0.4848	1
TAPPING_RA vs. TAPPING_LA	Unpaired	0.0279	0.0771	0.2000
REACTION_RA vs. REACTION_LA	Unpaired	1	0.5887	0.7000
NHPT_RA vs. NHPT_LA	Unpaired	0.0886	1	0.1000

RB, Right Before stimulation tDCS (right hand); RA, Right After stimulation tDCS (left hand); LB, Left Before stimulation tDCS (left hand); LA, Left After stimulation (left hand).

The appearance of statistically significant values after tDCS stimulation and mirror therapy was observed for the right (dominant) hand for the Reaction test in all *p* = 0.0371 (Table 4) and women *p* = 0.0313 (Table 4) and NHPT in all = 0.0273. These values indicate that tDCS stimulation and mirror therapy improved right hand function.

Furthermore, a statistically significant value was observed after stimulation with tDCS and mirror therapy for the right and left hand in the Tapping test u – all *p* = 0.0279, indicating that stimulation directly affected the function of the right hand and indirectly of the left hand.

## 4 Discussion

This study examined the impact of single session anodal tDCS on the left motor cortex, combined with mirror therapy, on the motor performance of both dominant and non-dominant hands in healthy individuals. We enrolled participants spanning three different age groups (40–49, 50–59, and 60–70 years). Overall, the results reported here seem to indicate that tDCS stimulation – applied over the motor cortex that represents the dominant hand – in conjunction with mirror therapy produced immediate improvements in speed, precision, and reaction time for the stimulated hand. Notably, a parallel enhancement was also observed for the non-dominant hand, indicating a possible interhemispheric transfer of motor benefits.

Our findings extend a growing body of literature showing that tDCS can increase motor function and motor learning in both healthy cohorts and clinical populations. Previous investigations have focused largely on post-stroke patients (Garrido et al., 2023; Bengisu et al., 2024), where multisession tDCS has proven beneficial for upper limb recovery (Seidel and Ragert, 2019). Studies in healthy adults have also reported that anodal tDCS over the primary motor cortex can strengthen motor performance and skill acquisition (Patel et al., 2019; Edwards et al., 2017). Here, we observed improvements after a single stimulation session, in line with research by Hazime et al. (2017), who demonstrated better isometric strength in healthy athletes after brief anodal stimulation (Hadi et al., 2021; Grosprêtre et al., 2021). Furthermore, the observed cross-limb benefits echo the results of Kaminski et al. (2022), who similarly reported cross-limb transfer when one hemisphere was targeted with tDCS during motor training. Our use of mirror therapy appears to have amplified these effects: by providing continuous visual input of the moving hand, mirror therapy may have enhanced bilateral sensorimotor network activation, facilitating plastic changes in both hemispheres (Zhao et al., 2022; Madhoun et al., 2020).

Mechanistically, the improvement seen in the non-dominant hand suggests involvement of interhemispheric communication. Anodal tDCS has been shown to increase cortical excitability, while mirror therapy promotes sensorimotor activation and top-down feedback by leveraging visual feedback from the moving limb (Madhoun et al., 2020; Grunt et al., 2017; Liron et al., 2012). The combined approach in this study may have optimized interhemispheric balance, such that the stimulated hemisphere not only facilitated motor responses in its contralateral limb but also modulated excitability in the opposite hemisphere. Such bilateral neuroplastic changes are well documented in stroke rehabilitation (Liao et al., 2020; Lerma-Lara et al., 2021; Lefebvre et al., 2014) and may similarly account for the generalized improvements observed among healthy participants in the current work.

One noteworthy aspect of our findings is that older participants (60–70 years) also showed benefits in motor testing. Previous research has been inconclusive on whether advanced age diminishes tDCS efficacy, partly because older adults may exhibit reduced plasticity or age-related cortical atrophy (Maudrich et al., 2022; Nyberg and Wåhlin, 2020; Coupé et al., 2019; Fjell et al., 2014; Lemaitre et al., 2012). However, our data indicate that even a single session of tDCS plus mirror therapy can significantly affect hand function in later life. Additionally, both men and women showed improvements, though certain comparisons revealed that men occasionally derived less benefit than women in specific metrics. This pattern mirrors the subtle differences reported in other tDCS research (Adenzato et al., 2019) and underscores the importance of analyzing sex-specific or gender-specific responses in future studies with larger subgroup samples.

Despite promising results, several limitations warrant caution. First, an important methodological limitation of our study is the use of a single-group, pre–post design without a sham tDCS or mirror-therapy-only control condition. As all participants received the combined intervention, we are unable to disentangle the specific contribution of transcranial stimulation vs. mirror therapy. While this design was selected to maximize within-subject statistical power in a proof-of-concept setting, it does not permit inference about the independent or interactive effects of each modality. Future studies should adopt a factorial design (tDCS alone, mirror therapy alone, both, and neither) to better clarify the mechanisms underlying the observed improvements. Nonetheless, our findings provide an empirical foundation for such trials by demonstrating that the combined intervention produces measurable and generalizable motor benefits even after a single session. Second, to maintain comparability we used identical pre- and post-stimulation tests; this choice raises the possibility of practice-related improvements independent of the intervention. Future studies should therefore establish baseline hand function after a longer interval or employ alternate task versions to minimize learning effects. Third, we used a single stimulation session. Longer intervention periods or multiple sessions of tDCS could potentially produce more robust or longer-lasting improvements, as suggested by multisession protocols in clinical contexts (Brunoni et al., 2016). Fourth, although we applied well-established outcome measures (e.g., Nine Hole Peg Test, Tapping Test, and Reaction Test), some of our digital tapping-based assessments (Click Speed Test and Flipflop Reflex Test) are less commonly used in peer-reviewed tDCS studies. While these measures provided easily quantifiable time data, broader usage of standardized or clinically validated metrics (e.g., Jebsen-Taylor Hand Function Test) might strengthen external validity. Fifth, our participants were stratified into decade-based age ranges (40–49, 50–59, and 60–70 years), a grouping that inevitably introduced some degree of interindividual variability—particularly within the oldest cohort, where age-related declines in cortical plasticity are known to vary substantially. While the sample size was adequate to detect main effects, future studies employing larger cohorts would enable more robust subgroup analyses (e.g., distinguishing between younger-old and older-old adults) and allow for a more nuanced examination of individual differences in responsiveness to tDCS. This decision to focus on middle-aged and older adults was further supported by findings from a pilot study involving participants aged 20–30 years, who completed the identical protocol (Wójcik et al., 2024). In that cohort, high baseline scores and a lack of statistically significant pre–post differences indicated a likely ceiling effect, suggesting limited potential for observable gains in younger individuals. Finally, it remains unclear how long the observed effects persist. Incorporating follow-up time points would help clarify whether single-session gains translate to lasting benefits in motor control. Future studies should systematically investigate several key areas.

## 5 Conclusion

In summary, our results highlight that a single 20-min session of anodal tDCS over the left motor cortex, paired with a brief period of mirror therapy, confers immediate gains in motor control for the dominant hand while also indirectly improving non-dominant hand performance across multiple age strata. These findings not only underscore the adaptability of the aging motor system, but also present a simple, non-invasive strategy to improve upper limb function in healthy individuals. By combining cortical neuromodulation with visual-sensory motor training, researchers and clinicians may open new pathways to optimize motor performance – both in rehabilitation and potentially even in high-performance contexts.

## Data Availability

The raw data supporting the conclusions of this article will be made available by the authors, without undue reservation.
